# mirnaQC: a webserver for comparative quality control of miRNA-seq data

**DOI:** 10.1093/nar/gkaa452

**Published:** 2020-06-02

**Authors:** Ernesto Aparicio-Puerta, Cristina Gómez-Martín, Stavros Giannoukakos, José María Medina, Juan Antonio Marchal, Michael Hackenberg

**Affiliations:** Department of Genetics, Faculty of Science, University of Granada, 18071 Granada, Spain; Bioinformatics Laboratory, Biotechnology Institute, Centro de Investigación Biomédica, PTS, Avda. del Conocimiento s/n, 18100-Granada. Spain; Instituto de Investigación Biosanitaria ibs.GRANADA, University of Granada, 18071 Granada, Spain; Excellence Research Unit "Modelling Nature" (MNat), University of Granada, 18071 Granada, Spain; Department of Genetics, Faculty of Science, University of Granada, 18071 Granada, Spain; Bioinformatics Laboratory, Biotechnology Institute, Centro de Investigación Biomédica, PTS, Avda. del Conocimiento s/n, 18100-Granada. Spain; Department of Genetics, Faculty of Science, University of Granada, 18071 Granada, Spain; Bioinformatics Laboratory, Biotechnology Institute, Centro de Investigación Biomédica, PTS, Avda. del Conocimiento s/n, 18100-Granada. Spain; Department of Genetics, Faculty of Science, University of Granada, 18071 Granada, Spain; Bioinformatics Laboratory, Biotechnology Institute, Centro de Investigación Biomédica, PTS, Avda. del Conocimiento s/n, 18100-Granada. Spain; Instituto de Investigación Biosanitaria ibs.GRANADA, University of Granada, 18071 Granada, Spain; Excellence Research Unit "Modelling Nature" (MNat), University of Granada, 18071 Granada, Spain; Department of Human Anatomy and Embryology, Institute of Biopathology and Regenerative Medicine, University of Granada, Granada, Spain; Department of Genetics, Faculty of Science, University of Granada, 18071 Granada, Spain; Bioinformatics Laboratory, Biotechnology Institute, Centro de Investigación Biomédica, PTS, Avda. del Conocimiento s/n, 18100-Granada. Spain; Instituto de Investigación Biosanitaria ibs.GRANADA, University of Granada, 18071 Granada, Spain; Excellence Research Unit "Modelling Nature" (MNat), University of Granada, 18071 Granada, Spain

## Abstract

Although miRNA-seq is extensively used in many different fields, its quality control is frequently restricted to a PhredScore-based filter. Other important quality related aspects like microRNA yield, the fraction of putative degradation products (such as rRNA fragments) or the percentage of adapter-dimers are hard to assess using absolute thresholds. Here we present mirnaQC, a webserver that relies on 34 quality parameters to assist in miRNA-seq quality control. To improve their interpretability, quality attributes are ranked using a reference distribution obtained from over 36 000 publicly available miRNA-seq datasets. Accepted input formats include FASTQ and SRA accessions. The results page contains several sections that deal with putative technical artefacts related to library preparation, sequencing, contamination or yield. Different visualisations, including PCA and heatmaps, are available to help users identify underlying issues. Finally, we show the usefulness of this approach by analysing two publicly available datasets and discussing the different quality issues that can be detected using mirnaQC.

## INTRODUCTION

Different aspects of miRNA-seq such as RNA extraction, storage conditions and sample processing together with the chosen library preparation protocol have a great impact on the obtained sequencing results (1,2). In any bioinformatics analysis of high-throughput sequencing data, quality control (QC) is the key step to reveal the existence of technical artefacts. Neglecting this step can lead to both false discoveries and failure to identify the existing biological signal. The processing of miRNA-seq data is no exception, and QC approaches should focus on measurable sample features that can be linked to quality aspects. Moreover, whenever possible, these quality parameters should hint or point out specific technical artefacts. This approach would offer the user the chance to take appropriate actions like excluding low-quality samples from the analysis or applying statistical models in order to correct for such technical variation when possible like in the case of batch effects (3).

Several sample attributes are generally calculated in sequencing experiments including the total number of sequenced reads, number of adapter-trimmed and filtered reads, percentage of mapped/unmapped reads and PhredScore to measure the quality of the sequencing. Besides these general statistics, many pipelines such as sRNAbench (4), mirTrace (5) or miRge (6) implement measurements that are specifically useful for miRNA-seq analysis like number of unique reads, percentage of miRNA-mapping reads, read length distribution and relative abundance of fragments from other RNA types (mostly tRNA and rRNA). Many of these parameters are clearly relevant for quality. Some indicate good quality samples when they hold high values (number of miRNA reads, total number of reads) while others (percentage of rRNA, adapter dimers) do it when they are low. Some of these features can be directly linked to a particular artefact, like a high percentage of adapter dimers which is normally caused by issues with adapters and/or input RNA concentrations (7). Other measurements, like smeared out read length distributions, can also be attributed to specific problems, in this case RNA degradation. However, most of them can be affected by several different artefacts thus it is frequently not possible to directly reveal specific technical issues when considering each quality feature individually. For instance, the yield in microRNAs can be influenced by any artefacts that impact the total read yield including contamination.

Regardless of the values good quality measurements should take, the context-free interpretation of sample features is generally not straightforward. For example, as an obvious source of unwanted fragments, rRNA presence should be minimized, but it is difficult and arbitrary to establish a specific threshold (5%, 10%, 20%) to discard samples. Therefore, rather than working with predefined or user-provided values that are hard to justify, a more agnostic approach would rely on relative values calculated from a background of comparable experiments (i.e. similar samples) which would in turn simplify the interpretation of the QC outcome.

A vast amount of publicly available data exists that can be exploited for purposes beyond their original goal (8). To generate a reference corpus of experiments that can be used to rank quality features, we downloaded over 36 000 raw sequencing datasets from the Sequence Read Archive (SRA) covering most model species. Samples were first processed using sRNAbench and then 34 quality features were extracted from each sample and subsequently organised into the reference set. Furthermore, sample metadata is used to tailor comparisons to more relevant sets of experiments (i.e. samples from the same species and/or processed with the same library preparation protocol).

In contrast with previously available software (5,6), mirnaQC calculates absolute and relative values for several quality-related features for a set of miRNA-seq samples. Input data can be uploaded as FASTQ files or provided as SRA run accessions that will subsequently be ranked making use of the reference corpus mentioned above. An apparent advantage of this approach is that fixed thresholds are no longer needed and decisions can be made based on background statistics. Users can explore mirnaQC results by means of interactive plots and tables that hold both absolute and relative values of the 34 quality attributes. The output report is structured into several categories trying to relate the quality attributes to the different possible technical artefacts. This approach can help to identify low quality samples or reveal issues in the sample processing which is extremely important for protocol optimisation.

## mirnaQC SAMPLE FEATURES AND QUALITY MEASURES

The success of a small RNA sequencing run depends on many different factors including RNA quality, quantity and purity, an optimized library processing protocol and the sequencing itself. However, it is not always easy or even possible to directly relate features extracted from sequencing data to any technical artefacts. mirnaQC calculates and ranks several quality parameters conceived to hint problems in the different aspects involved in the preparation of miRNA sequencing libraries. Below we describe the different sections and, wherever possible, the putative artefacts or quality issues that can be derived from them.

### Sequencing yield

This section focuses on the amount of reads and the fraction that can be assigned to known miRNAs. Generally, parameters in this category (percentage of valid reads, detected microRNAs) indicate high quality when they hold high values. Low numbers (especially for the percentage of valid input reads) can be related to problems in RNA processing or low input material. Some sources like exosomes extracted from bodily fluids however, are known to hold low levels of miRNA, thus high numbers should not be expected for all sample types even for high quality libraries.

### Library quality

In this category we list the number of reads that are filtered out due to minimum length (15nt), the percentage of ribosomal RNA and the percentage of short reads (15–17 nt). Their presence may be attributed to degradation products from longer RNA molecules as no small RNAs are known in this length range.

High percentages of adapter-dimers (0, 1 or 2 nt fragments after trimming) normally indicate issues with the ratio of adapter to input RNA concentration. In practice, it is very difficult to completely avoid adapter-dimers, especially in low input samples such as blood. Nevertheless the percentile may still be useful as it might show potential for improvement.

Ultra-short reads are defined as fragments with lengths between 3nt and 14nt (both inclusive).

### Library complexity

In general it is also interesting to assess the complexity of the sample since low complexity libraries provide very little information, even for otherwise high-quality datasets. Several measurements are provided to grasp the complexity at two levels that should be interpreted together:

- Sequencing library complexity: This is calculated as the ratio of the total number of reads to unique reads. Lower values suggest higher RNA diversity but it can also be caused by degradation.
- miRNA complexity: Frequently most microRNA reads correspond to few miRNA genes preventing lowly expressed miRNAs from being detected. Several measures are given to estimate complexity at this level: (i) percentage of miRNA expression assigned to the first, the first 5 and first 20 most expressed miRNAs, (ii) the number of miRNAs required to reach 50%, 75% and 95% of the total miRNA expression.

### Putative contamination

The percentage of reads that could not be mapped to the species’ genome is calculated. Contamination is subsequently estimated by mapping against a collection of bacterial and viral genomes.

### Read length distribution

A narrow peak around 22 nucleotides in the read length distribution indicates good quality samples whereas degraded or poor RNA quality manifests in a broader distribution. Furthermore, it is clear that the 22nt peak should be present for miRNA assigned reads and RNA quality issues might exist if samples deviate from this.

We summarise the miRNA read length distribution in several ways: mean length, mode of the distribution, the fraction of reads with lengths 21, 22 or 23 nt, the standard deviation and the skewness of the distribution.

### RNA composition

The relative abundance of other RNA molecules is automatically profiled using the sRNAtoolbox database (9). Most of these longer RNA species (rRNA,mRNA, lincRNA) are not known to be processed into smaller molecules that can be picked up by miRNA-seq. Their presence is a symptom of RNA degradation since smaller fragments are randomly generated and then sequenced. Among these, rRNA is typically used because it's the most abundant one.

### Sequencing quality

Sequencing quality is calculated by means of FastQC (10). We determine the mean values of the different percentiles provided by the program over all positions of the read.

## GENERATION OF THE miRNA-seq REFERENCE CORPUS (BACKGROUND KNOWLEDGE)

The vital part of the presented quality control tool mirnaQC is the comparison corpus of miRNA-seq data that is used to rank user's samples. Using OmicIDXR API we obtained a list of >3000 SRA studies that were annotated as ‘miRNA-Seq’ or ‘ncRNA-Seq’ (several ‘RNA-Seq’ were also included after checking they were in fact ‘miRNA-Seq’ datasets).

For each study we performed the following steps:

- Read the meta-data for a study generating one entry per experiment (SRX level)
- Download all SRR files that correspond to this SRX by means of fastq-dump (fastq.gz)
- Detect the library preparation protocol
- Analyse the small RNA sequencing data with sRNAbench using all available annotations from sRNAtoolboxDB
- Upload sRNAbench results to a MySQL database

In total we analysed 36 338 samples from 30 different species. We distinguish 8 different protocols: Illumina, Illumina_2 (3′ adapter sequence), Next England Biolabs (NEBnext), Qiagen_UMI, NextFlex, adapter trimmed, SoliD and all others (custom). Over 500 billion sequencing reads were analysed.

## mirnaQC WORKFLOW AND IMPLEMENTATION

**Figure 1.**
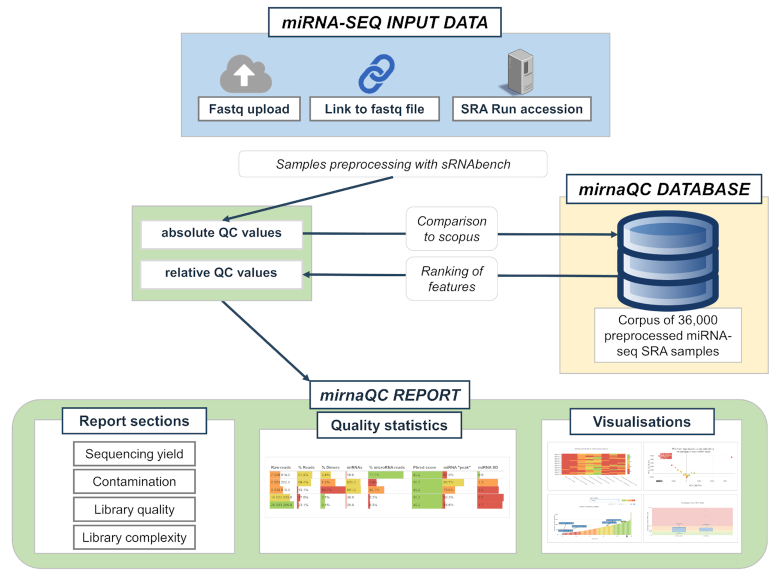
A schema of the front end and workflow of mirnaQC. Some features of the quality report are depicted at the bottom.

An overview of the mirnaQC workflow is displayed on Figure 1. The only required input is sequencing data in FASTQ format (or SRR accessions) although sample species and library protocol information is recommended if known. If the protocol or species are not provided by the user an automatic detection algorithm, trained with a set of manually curated samples from liqDB (11), will find the right input parameters. Condition or group information can also be provided (optional). All files belonging to a given group should be compressed into a single *.zip, tar.gz* or *.7z* file and then separately uploaded. The file names will be used as group labels, and this information will appear in some of the plots.

Input data is subsequently processed by sRNAbench in two steps: First reads are simultaneously mapped to the species genome and a collection of virus and bacterial genomes from sRNAtoolboxDB (9) allowing one mismatch. Preference is given to the reference genome in case of multiple mapping reads. In the second step reads are mapped to microRNA reference libraries (12,13), RNAcentral (14) and Ensembl annotations (15) for ncRNA and mRNA. Note that although samples are mapped to both reference libraries, miRBase and MirGeneDB, currently the miRNA related figures are extracted from the miRBase mappings.

Out of both sRNAbench output folders we extract a total of 34 quality attributes that are next compared to 5 different reference sets: (i) samples from the same kingdom (animals and plants), (ii) samples from the same species, (iii) samples from the same kingdom and protocol, (iv) samples from the same species and protocol and (v) low-input samples (defined as those obtained from bodily fluids). Each comparison can be browsed separately on the output page.

**Figure 2.**
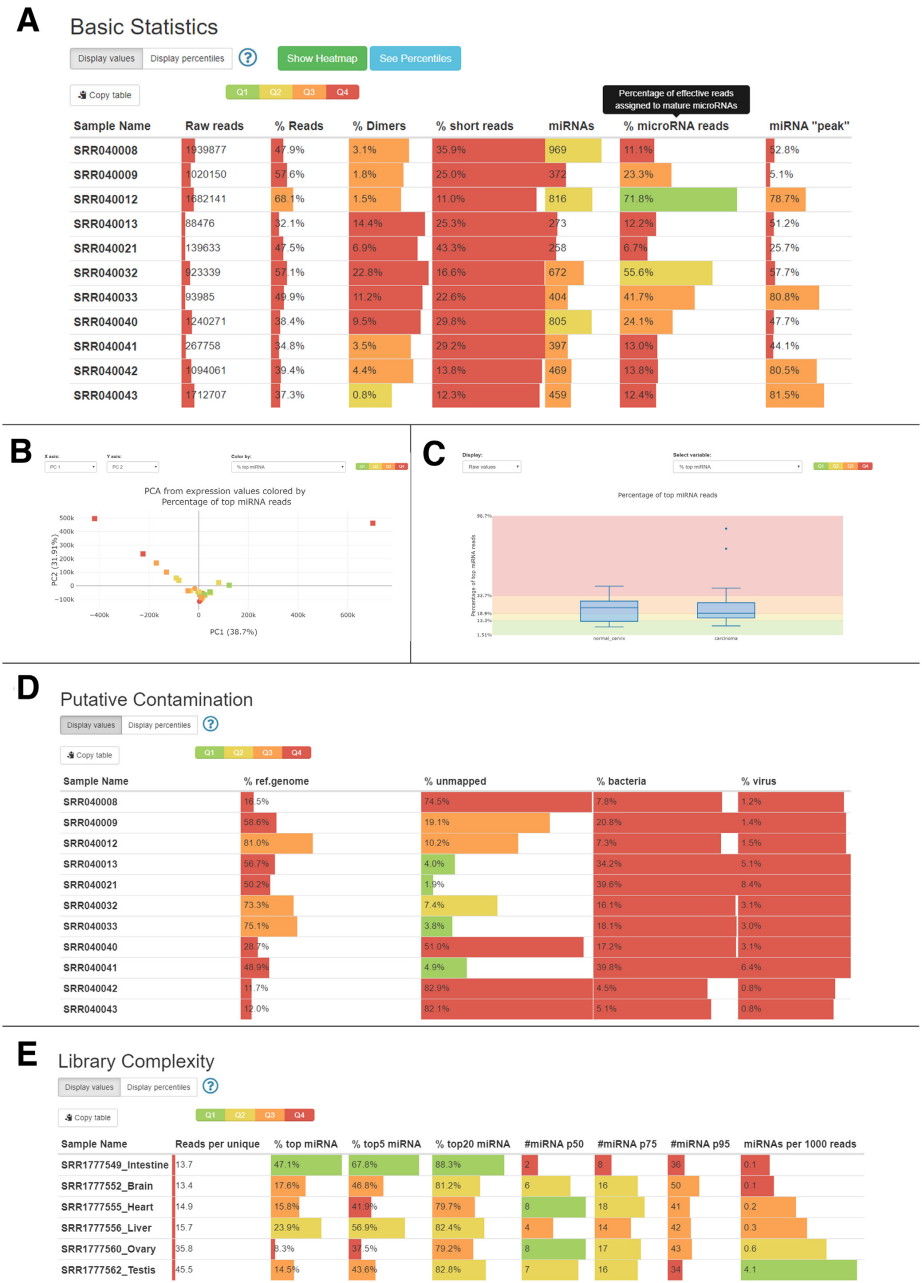
Different examples of mirnaQC sections and visualisations. (**A–D**) display different quality aspects of a cancer study. (**E**) shows the output of different sample complexity measures for different tissue types and two columns from Basic Statistics section at the right.

The processing pipeline is a java programme that includes sRNAbench, Bowtie (16) and a MySQL client to query the reference corpus. The web interface was developed using Python and Django and runs on Apache. The results report includes the six sections described in the *mirnaQC sample features and quality measures* with tables and styles from multiQC (17) and Plotly visualisations. Both absolute values and percentiles are displayed and highlighted using a quartile colour code (see Figure 2C).

## WORKING EXAMPLE

To show the usefulness of this tool, we analysed two publically available studies. Basic statistics from the first dataset, one of the earliest large studies designed to detect cervical cancer (18), can be seen on Figure 2A. To help users identify potential issues, the quality parameters and their percentiles are displayed using a quartile-based colour code (from better to worse values: green, yellow, orange and red). Using this guide, several problems can be identified: with few exceptions, most parameters rank on the third (orange, Q3) and fourth quartiles (red, Q4). More specifically, miRNA ‘peak’ values show that a rather low percentage of microRNA reads have lengths between 21 and 23nt in the majority of samples. This means that although those reads can be assigned to miRNA reference sequences, they do not correspond to the canonical miRNA lengths. This hints an RNA processing issue that might still be tolerated if all samples are similarly affected, which can indicate either systematic artefacts or biological reasons.

It may also happen that not all samples are equally affected by a quality issue, which can be more problematic if two or more conditions are to be compared. mirnaQC allows users to assign samples to conditions in order to explore this possibility. Figure 2B shows a PCA plot of the expression values of the 50 most expressed miRNAs. Users can decide which quality attribute should be used to colour the markers, in this case we used ‘% top miRNA’ (the percentage of reads assigned to the most abundant microRNA). This graph shows that the two outlier samples are much less complex than the rest. Furthermore, because conditions are marked with different symbols (control-circles and carcinoma-squares), we know that these two samples belong to the same group. Keeping such samples in the analysis is not recommended since they will certainly bias the results. Figure 2E displays the distribution of this feature for both groups by means of boxplots. Here we can see that these two samples are outliers but otherwise both conditions show reasonably similar distributions for this parameter.

Users can also explore potential sources of contamination from reads mapped to viral and bacterial genomes. Figure 2D shows that all samples suffer from rather high percentages of contamination reads. All samples have more bacterial/viral reads than 75% of all animal samples in the reference set. This range of values indicates serious contamination with the possible exception of cervical cancer samples, where this might be caused by sample extraction or even have a role in the disease (19).

Finally, Figure 2E shows the library complexity of different tissues from *Takifugu rubripes* (20). While the top expressed microRNA in intestine picks up 47.1% of all reads (percentile 88.4), in ovary this figure drops to 8.3% which corresponds to percentile 2.6. In ovary, eight microRNA sum up over 50% of all miRNA expression while in intestine it takes only two to reach the same percentage which indicates a higher complexity of miRNA expression in germ cells. Furthermore, ovary and testis exhibit much lower percentages of miRNAs. This might be related to their larger repertoire of small RNAs in germ cells (21) which automatically would lead to a lower relative fraction of microRNAs in those samples.

## CONCLUSION

We present a user-friendly web server for the comparative quality control of miRNA-seq data that can be useful in several scenarios: to identify low-quality samples that should be excluded from downstream analysis; to reveal systematic errors in order to improve the library preparation process, something especially relevant for pilot studies; and finally, to provide external quality validation for datasets so it can be used as a standard proof of quality.

mirnaQC provides several output tables and visualisations for a total of 34 quality attributes which allow users to rank their results against a large corpus of comparable samples. In this way, no absolute thresholds need to be applied and the user can evaluate their sequencing data based on percentiles. Future developments include new types of analysis and improved visualisations intended to detect confounding variables related to quality issues that can affect downstream steps. Additionally, a dockerized version of the tool will be made available so the pipeline can be run locally or in computing clusters.

## DATA AVAILABILITY

https://arn.ugr.es/mirnaqc/.
